# Call for Participation: Collaborative Benchmarking of Functional-Structural Root Architecture Models. The Case of Root Water Uptake

**DOI:** 10.3389/fpls.2020.00316

**Published:** 2020-03-31

**Authors:** Andrea Schnepf, Christopher K. Black, Valentin Couvreur, Benjamin M. Delory, Claude Doussan, Axelle Koch, Timo Koch, Mathieu Javaux, Magdalena Landl, Daniel Leitner, Guillaume Lobet, Trung Hieu Mai, Félicien Meunier, Lukas Petrich, Johannes A. Postma, Eckart Priesack, Volker Schmidt, Jan Vanderborght, Harry Vereecken, Matthias Weber

**Affiliations:** ^1^Institut für Bio- und Geowissenschaften: Agrosphäre (IBG-3), Forschungszentrum Jülich GmbH, Jülich, Germany; ^2^International Soil Modelling Consortium ISMC, Jülich, Germany; ^3^Department of Plant Science, The Pennsylvania State University, University Park, PA, United States; ^4^Earth and Life Institute, Agronomy, Université Catholique de Louvain, Louvain-la-Neuve, Belgium; ^5^Institute of Ecology, Leuphana University Lüneburg, Lüneburg, Germany; ^6^INRAE, Avignon Université, EMMAH, Avignon, France; ^7^Earth and Life Institute, Environmental Sciences, Université Catholique de Louvain, Louvain-la-Neuve, Belgium; ^8^Department of Hydromechanics and Modelling of Hydrosystems, University of Stuttgart, Stuttgart, Germany; ^9^Simulationswerkstatt, Leonding, Austria; ^10^CAVElab–Computational and Applied Vegetation Ecology, Ghent University, Ghent, Belgium; ^11^Department of Earth and Environment, Boston University, Boston, MA, United States; ^12^Institute of Stochastics, Ulm University, Ulm, Germany; ^13^Institut für Bio- und Geowissenschaften: Plant Sciences (IBG-2), Forschungszentrum Jülich GmbH, Jülich, Germany; ^14^Institute of Biochemical Plant Pathology, Helmholtz Zentrum München, Neuherberg, Germany

**Keywords:** functional-structural root architecture models, model comparison, benchmark, root water uptake, call for participation

## Abstract

Three-dimensional models of root growth, architecture and function are becoming important tools that aid the design of agricultural management schemes and the selection of beneficial root traits. However, while benchmarking is common in many disciplines that use numerical models, such as natural and engineering sciences, functional-structural root architecture models have never been systematically compared. The following reasons might induce disagreement between the simulation results of different models: different representation of root growth, sink term of root water and solute uptake and representation of the rhizosphere. Presently, the extent of discrepancies is unknown, and a framework for quantitatively comparing functional-structural root architecture models is required. We propose, in a first step, to define benchmarking scenarios that test individual components of complex models: root architecture, water flow in soil and water flow in roots. While the latter two will focus mainly on comparing numerical aspects, the root architectural models have to be compared at a conceptual level as they generally differ in process representation. Therefore, defining common inputs that allow recreating reference root systems in all models will be a key challenge. In a second step, benchmarking scenarios for the coupled problems are defined. We expect that the results of step 1 will enable us to better interpret differences found in step 2. This benchmarking will result in a better understanding of the different models and contribute toward improving them. Improved models will allow us to simulate various scenarios with greater confidence and avoid bugs, numerical errors or conceptual misunderstandings. This work will set a standard for future model development.

## 1. Introduction

A growing number of different modeling techniques and software libraries are now available to build functional-structural root architecture models. Different available models of root architecture and functions have been discussed and qualitatively compared in Dunbabin et al. (2013). The available models differ in the way they represent different processes, such as root growth, water flow, solute transport are captured and translated into mathematical equations (process-level differences); in how they solve mathematical problems by their choice of analytical or numerical approach, numerical scheme, programming technique (solution-level differences); and in how they couple the different processes to the full model (coupling-level differences). However, the extent of discrepancies is currently unknown. Thus, a framework for quantitatively comparing functional-structural root architecture models is required. In addition to the explanatory or predictive power of a model, it is also important to understand the performance of these models, e.g., in terms of accuracy or computational cost. The most commonly used type of functional-structural root architecture models represent the structure of the root system as a 1-dimensional branched network of discrete segments which is geometrically embedded in a 3-dimensional soil domain (Koch et al., 2018b). The root architecture may either be known from measurements, such as 2D or 3D images, or from root architectural models. Suitable models are then used to simulate the “functions,” such as carbon flow and use in root systems (e.g., Bidel et al., 2000), rhizodeposition (Nygren and Perttunen, 2010), competition between species (Dunbabin, 2007), plant anchorage (Dupuy et al., 2007), water and nutrient uptake (Dunbabin et al., 2006; Javaux et al., 2008). Exchange between soil and root is typically modeled via source/sink terms. From the point of view of the soil domain, roots are often considered as line sources, i.e., it is assumed that their diameter is small compared to the relevant spatial scale of the soil. The advantage of this approach is that it does allow to consider root system architecture (position of each segment in time and 3D space) explicitly while being computationally less expensive than an explicit representation of root volumes in the soil domain. By direct comparison with explicit 3D simulations, Daly et al. (2018) showed for the case of young wheat plants that the error made by neglecting root volumes physically present in the soil domain is negligibly small in case of root water uptake. Thus we may expect that, for plants where the line source assumption holds, models of this type are sufficiently accurate. They are also computationally cheaper than explicit 3D and allow the consideration of older and thus larger root systems. The challenge is now to develop a commonly accepted framework for benchmarking functional-structural root architecture models. This includes defining a set of benchmark problems to test model accuracy and performance. We propose that models should be evaluated against two different kinds of references: First, we will develop simple benchmark scenarios, if possible with analytical solutions, that serve as a reference for model verification. Secondly, we define data sets that can be used as references for the evaluation of more complex models without analytical solution. These data sets should as good as possible describe the system we want to model and contain as little uncertainty as possible (Luo et al., 2012). This benchmark activity focuses on two processes, root architecture development and root water uptake. We propose this benchmarking framework to be used by the community of modelers and other participants to compare their model outputs against those of the reference solutions of benchmarks defined in this paper. The use of this framework thus aims to be a collaborative effort. We will refer to any numerical model that implemented some or all of the benchmark problems as “participating model” or “simulator.”

## 2. Benchmark Problems for Models of Root Architecture and Function

In order to benchmark models of root architecture and function, we propose a multi-step approach with growing level of complexity. The individual benchmarks refer as much as possible to published work, however, we streamlined the different problems and made the notation consistent throughout this paper. A list of symbols is provided in Table 1. The intrinsic nature of functional-structural root architecture models involves multiple coupled domains and processes. A single process in a single domain (e.g., water flow in soil) is referred to as “module” here. The first set of benchmarks (M1–M3) is about individual modules (M) only, i.e., they either deal with only root growth, water flow in soil or water flow inside roots. The scenarios are simple, possibly have analytical solutions, and the goal is to build trust in the accuracy of the individual participating models and to help interpret potentially diverging results of the coupled benchmark problems. Benchmark problems M1 are about root architecture development. It is known that the representation of growth processes can be very different between different simulators. Thus, the goal is to calibrate each simulator individually to given root image data (reference data). M2 is about modeling water flow in soil. Here all participating models solve the same equation, namely the Richards equation, and differences may occur due to differences in numerical implementation. M3 deals with water flow inside the root system for static soil water conditions. As for M2, differences between models are expected to be mainly due to the numerical implementation of this well-defined process. The second set of benchmarks (C1 and C2) is about coupled root-soil models. Benchmark problems C1 consider a static (non-growing) root system and focus on comparison of numerical representation of agreed-upon equations and process representations as well as on the coupling approach to compute the sink term for root water uptake. For this benchmark, we provide a reference solution that is based on a computational mesh that was generated with consideration of the physical presence of the roots in the soil domain. Thus, root water uptake was simulated not by a sink term but as a boundary condition at the root surface in soil. Our approach is similar to Daly et al. (2018) but in addition couples the soil domain to the root domain so that pressure gradients along the roots are simulated. Benchmark problem C2 compares the water uptake of fully coupled models with growing root systems.

**Table 1:** List of notations.

**Symbol**	**Units**	**Description**
*d*	cm	Depth
*D*_*w*_	cm^2^d^−1^	Water diffusivity
**e_3_**	(0,0,1)	Standard unit vector
*J*	cm^3^cm^−2^d^−1^	Water flux per unit soil surface area
*k*_*r*_	cm^3^cm^−2^cm^−1^d^−1^	Root radial conductivity (defined as volume of water per unit root surface area, pressure head gradient and time)
*k*_*x*_	cm^4^cm^−1^d^−1^	Specific root axial conductance
*K*(θ)	cm^3^cm^−2^d^−1^	Soil hydraulic conductivity
*K*_*sat*_	cm^3^cm^−2^d^−1^	Saturated soil hydraulic conductivity
*l*	cm	Length
*n*	–	van Genuchten shape parameter
*q*	cm^3^cm^−2^d^−1^	Water flux per unit root surface area
*Q*	cm^3^d^−1^	Volumetric water flow rate
$\bar{Q}$	cm^3^d^−1^	Daily average volumetric water flow rate
*Q*_*r*_	cm^3^d^−1^	Radial root water flow rate
*Q*_*x*_	cm^3^d^−1^	Axial root water flow rate
*r*_*root*_	cm	Root radius
*S*_*w*_	cm d^−0.5^	Sorptivity (infiltration) or desorptivity (evaporation)
*t*	d	Time
**v**	(*v*_1_,*v*_2_, *v*_3_)	Normalized direction of the xylem, pointing toward the root tip
*w*	cm	Width
*x*, *y*, *z*		Spatial coordinates, z-axis pointing upward, soil surface is at *z* = 0
*Y*	–	Cumulative root fraction from surface to depth *d*
α	cm^−1^	van Genuchten shape parameter
β	–	Root distribution index
η	cm	Position of the infiltration front (Equation 4)
λ	–	van Genuchten-Mualem parameter
Λ	–	Root domain (network of root center-lines)
Ω	–	Soil domain
Φ	cm^2^d^−1^	Matric flux potential
θ	cm^3^cm^−3^	Volumetric water content
θ_*a*_	cm^3^cm^−3^	Reference water content
θ_*res*_	cm^3^cm^−3^	Residual water content
θ_*sat*_	cm^3^cm^−3^	Saturated water content
ψ	cm	Water pressure head, described as potential energy per unit weight of water (i.e., units are cm of water column), given as relative to air pressure of 1,020 cm and excluding the gravitational potential
ζ		Local coordinate along root axis
**Sub indices**		
collar	Root collar (upper boundary of root system domain)	
i	Initial	
pot	Potential	
r	Radial	
res	Residual	
s	Soil	
sat	Saturation	
seg	Root segment	
sim	Simulation	
sur	Soil surface	
tip(s)	Root tip(s) (boundaries of root system domain)	
top	Top, position of the soil surface	
out	Outer radius of soil cylinder around a single root	
x	Xylem	

Each benchmark problem is described in a Jupyter Notebook that is publicly available on a github repository. Each Jupyter Notebook has a list of contributing authors at its beginning. We will provide codes for automatic analyses and comparison of different model results with the reference solutions or reference data. This makes the analysis transparent and easily modifiable and facilitates including even future participating models' outputs at any later time.

### 2.1. Levels of Contribution

Any group using or developing functional-structural root architecture models is invited to participate in this collaborative model comparison. Not every model might be suited for all of the provided benchmark problems. Thus, every participant may decide in which individual benchmark problem they would like to participate. However, to reach a certain level of complexity, the “module” benchmarks should be simulated first before the “coupled” benchmarks. Table 2 gives an overview of the key features of these problems and their implementations. One important aim of this activity is a joint publication that shows and discusses the results of the different participating models in comparison to the reference solutions and reference data provided as well as to gain an overview of the extent of deviations between the different simulators.

**Table 2:** Description of benchmark scenarios to be implemented in 3D functional-structural root architecture models^a^.

	**Benchmark problem**	**Domain**	**Initial conditions**	**Boundary conditions**	**Evaluation**	**Remarks**
RSA	M1.1: RSA calibration	*t*_*sim*_ = 11 (8) for lupine (maize)	Seed position (0,0,−3)	n.a.	Comparison against the measured root systems provided—traits and persistent homology (PH)	Model parameters are determined from calibration against traced images provided in the github repository in RSML format in the folder in M1.1 RSA calibration/M1.1 Reference data; 100 realizations for each model setup
	M1.2: RSA simulation	*t*_*sim*_ = 60	Seed position (0,0,−3)	n.a.	No reference solution, comparison amongst models—traits, PH, RLD	RSA model parameters from M1.1; 10 realizations for each model setup
Soil	M2.1: Infiltration	l × w × d = 10 × 10 × 200, *t*_*sim*_ = 1	ψ_*s,i*_ = −400	at $\begin{array}{l} z=0\{\begin{array}{l} J_{s}=-100\text{ if}\psi_{s}<0\\ \psi_{s}=0\text{ else} \end{array}\;,\\ \frac{\partial \psi_{s}}{\partial z}{|}_{z=200}=1 \end{array}$, no-flux at the sides	Analytical solution, Equation (4)	Sand, loam, clay (Table 3)
	M2.2: Evaporation	l × w × d = 10 × 10 × 100, *t*_*sim*_ = 10	ψ_*s,i*_ = −40 for sand and −200 for all other scenarios	at $z=0\{\begin{array}{l} J_{s}=J_{s,ref}\text{if }\psi_{s}>-10,000\\ \psi_{s}=-10,000\text{ else} \end{array}\;,$ no-flux at all other boundaries	Analytical solution, Equation (5)	Scenario 1: sand,*J*_*s,ref*_ = 0.1, scenario 2: loam,*J*_*s,ref*_ = 0.1, scenario 3: loam,*J*_*s,ref*_ = 0.3, scenario 4: clay,*J*_*s,ref*_ = 0.3
Xylem	M3.1: Single root	1 vertical root, L = 50	n.a.	ψ_*x*_|_collar_ = −1000, *Q*_*x*_|_tip_ = 0	Analytical solution, Equation (7)	*k*_*x*_ = 0.0432, *k*_*r*_=1.73 × 10^−4^, ψ_*s*_ = −200
	M3.2: Root system	14-days old root system	n.a.	ψ_*x*_|_collar_ = −500, *Q*_*x*_|_tips_ = 0	Hybrid analytical solution (Meunier et al., 2017)	Root hydraulic properties in scenario (a): Table 4, (b): Figure 7, ψ_*s*_ = −200, static RSA given in the root_grid folder of this benchmark
Coupled 1	C1.1: Single RWU	1D radially symmetric, *r*_*root*_ = 0.02, *r*_*out*_ = 0.6, *t*_*sim*_ = 20	ψ_*s,i*_ = −100	at $r=r_{root}\{\begin{array}{l} q_{r}=q_{root},\text{if}\psi_{s}>-15,000\\ \psi_{s}=-15,000\text{ else} \end{array}\;q_{r}{|}_{r=r_{out}}=0$	Analytical solution, Equations (11) and (12)	Sand, loam, clay (Table 3), scenarios 1–3: *q*_*root*_ = 0.1, scenarios 4–6: *q*_*root*_=0.05
	C1.2: RWU, static RSA	static 8-days old root system, soil: l × w × d = 8x8x15, *t*_*sim*_ = 3	ψ_*s,i*_ = −659.8−*z*	$\begin{array}{l} \{\begin{array}{l} Q_{x}{|}_{collar}=6.4\text{if}\psi_{x}{|}_{collar}>-15,290\\ \psi_{s}=-15,290\text{ else} \end{array}\;,\\ Q_{x}{|}_{tips}=0, \end{array}$no-flux at all soil faces	Reference solution: explicit 3D simulation	Loam (Table 3), static RSA given in the root_grid folder of this benchmark, root hydraulic properties in scenario (a): Table 4, (b): Figure 7
Coupled 2	C2.1: RWU, dynamic RSA	Growing root system, soil: l × w × d = 25 × 25 × 100, *t*_*sim*_ = 60	ψ_*s,i*_ = −200	$\{\begin{array}{l} Q_{x}=0.5\cdot rel_{LAI}\text{if }\psi_{x}>-15,000\\ \psi_{x}{|}_{collar}=-15,000\text{ else} \end{array}\;,Q_{x}{|}_{tips}=0,$no-flux at all soil faces	No reference solution, comparison amongst models	Loam (Table 3), *k*_*x*_=0.0432, *k*_*r*_ = 1.73 × 10^−4^, RSA parameters from M1.1, *rel*_*LAI*_ scales the potential transpiration

a *All paths are relative to the github repository https://github.com/RSA-benchmarks/collaborative-comparison.git. For other abbreviations and units see Table 1*.

#### 2.1.1. How to Participate

The participation includes three steps:

1. **Registration**: Any interested researcher is welcome to contact the corresponding author of this paper, Andrea Schnepf, with the following information: Name, affiliation, name or reference to the participating simulator. Upon signing a letter of agreement confirming that results of other participants will not be published without consent, researchers will be accepted as participants and enabled to include their individual simulation results to the github repository of this benchmark initiative, https://github.com/RSA-benchmarks/collaborative-comparison.
2. **Simulation**: Each participant implements all or a selected number of benchmark problems in their respective simulator and makes the results in the prescribed formats available to the github repository through pull requests. Requested formats include the Root System Markup Language, RSML (Lobet et al., 2015) for root architectures and the Visualization Toolkit, VTK (Schroeder et al., 2006) for 3D and 1D simulation outputs. Python scripts to read and write RSML files will be provided on the github repository. Packages to read and write VTK files are for example available at https://pypi.org/project/vtk/.
3. **Analysis and publication**: The analysis of results and computation of relevant metrics, such as root mean square error, coefficient of determination or Nash–Sutcliffe efficiency, will be done by the code implemented in the Jupyter Notebooks for each benchmark problem. The final goal is to jointly publish the results.

### 2.2. Benchmarks for Individual Modules

#### 2.2.1. Module M1: Root System Architecture Models

Root system architecture models (RSA models) are that module within a complex functional-structural plant model that simulates the structure, topology, and 3D placement of the roots. They simulate the growth of root systems as (upside down) tree-like structures based on rules regarding elongation, branching and death. Mostly, they are discrete models and represent the root system by a mathematical graph (i.e., nodes and edges/root segments). Each node or segment may be additionally associated with attributes, such as radius, age or hydraulic properties.

**Figure 1:**
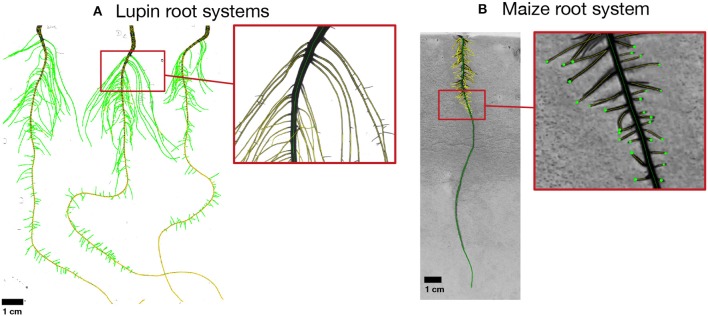
Example of root images used for the benchmarking dataset. **(A)** Shows an image of lupin root systems, 11 days old, growing in an aeroponic setup. **(B)** Shows an image of a maize root system growing on filter paper (5 days old). All images were analyzed using the semi-automated root image analysis software SmartRoot (Lobet et al., 2011), colors distinguish different root orders. The RSML files containing the full information about the root systems are provided on the github repository in the folder “M1.1 RSA calibration\M1.1 Reference data.”

**Figure 2:**
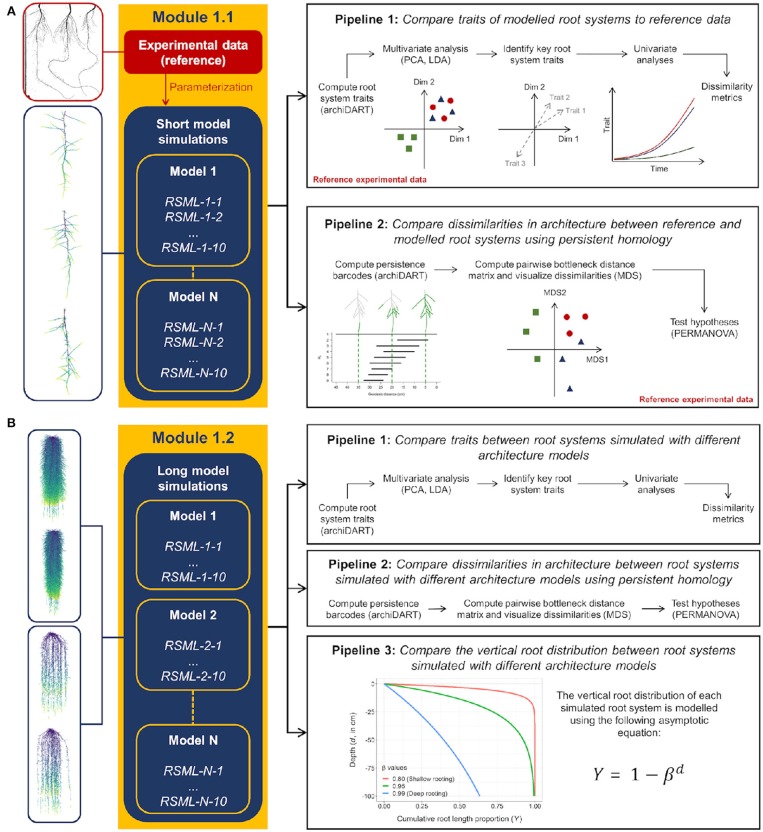
Presentation of the data analysis pipelines used for the benchmarking of root architecture models. **(A,B)** Show the first (M1.1) and second (M1.2) benchmark scenarios, respectively.

The aim of this first benchmarking exercise is to determine if root architecture models currently available are able to reproduce realistic root architectures when being parameterized on the basis of a common experimental data set (Figure 2A). The particular challenge to benchmark RSA models is to include the stochastic nature of these models. We propose to perform the benchmarking of those models in four steps: (1) Parameterizing the root architecture models based on the provided experimental data, (2) Simulating a set of root systems for a dicotyledonous (*Lupinus albus*) and a monocotyledonous (*Zea mays*) plant species following two benchmark scenarios (M1.1 and M1.2), (3) Export and store the simulated root systems as Root System Markup Language (RSML) files (Lobet et al., 2015), and (4) Compare the simulation results using the data analysis pipelines available in the associated Jupyter Notebooks. The analysis pipelines are explained below and illustrated in Figure 1. In particular, we include persistent homology as an approach that augments purely trait-based comparisons, i.e., two root systems with the same total root length could be very different based on the persistent homology approach.

##### 2.2.1.1. M1.1 Root system architecture model calibration

The different available root architecture models (see e.g., Dunbabin et al., 2013) are partly different in the way they represent the growth processes, such that the equations describing these processes are very different. For example, branch emergence is a function of apical zone length in CRootBox (Schnepf et al., 2018b) while it is a function of delay time in Root Typ (Pagès et al., 2004). Root radius is a time-dependent function scaled to distance along the root in OpenSimRoot (Postma et al., 2017) while it is computed according to the pipe model in ArchiSimple (Pagès et al., 2014). Thus, we are looking at process-level differences between the different models, and each participating RSA model will have a different set of parameters that drive root growth. This is the reason why, in this benchmark, we do not prescribe a parameter set as in e.g., M2 or M3, but we let each participating model derive its respective model parameters based on a reference dataset. In this first benchmark (M1.1), modelers simulate root systems for the same duration as the age of the root systems in the reference dataset.

*2.2.1.1.1. Reference data set.* Although the parameterization of 3D models using a set of parameters derived from 2D images has some limitations, it has been shown to be a simple and efficient strategy allowing the simulation of realistic 3D root systems (Landl et al., 2018). Our reference dataset contains two distinct sets of images: (1) images of lupin roots grown for 11 days in an aeroponic setup (Lobet et al., 2011), and (2) images of maize roots grown for 8 days on filter papers (Hund et al., 2009). All images were analyzed using the semi-automated root image analysis software SmartRoot (Lobet et al., 2011) and root tracings were saved as RSML files for further analysis (Figure 1). These RSML files were then processed using functions of the R package archiDART developed to compute root system- and single root-level metrics (Delory et al., 2016, 2018). These metrics have been made open-access (https://github.com/RSA-benchmarks/collaborative-comparison/tree/master/root_architecture/data) and should help modelers to parameterize their respective RSA model.

*2.2.1.1.2. Required output*. The following results are to be uploaded via pull requests to this path on the github repository: M1 Root architecture development/M1.1 RSA calibration/M1.1 Numerical results.

1. A text file including the outcome of the calibration step, i.e., the set of model input parameters required for the specific simulator.
2. Simulation output from running the root architecture model using this parameter set in RSML format. Due to the stochastic nature of root architecture models, 100 realizations of each model setup are requested. The file format should be RSML and the file name should be of the form “modelname_replicate,” e.g., “CRootBox_1.rsml.”

*2.2.1.1.3. Reference data analysis and automated model comparison*. Statistical evaluation of a root architecture model has for example been done by Delory et al. (2018); Schnepf et al. (2018a). This motivated the creation of two data analysis pipelines for the first benchmark (M1.1) that will be used to compare simulation outputs with reference experimental data (reference root systems) (Figure 2A). These two data analysis pipelines are implemented in the Jupyter Notebook RSA calibration.ipynb that can be found on the github repository that contains code that will automatically include every model output in the analysis that is available in the prescribed folder. The analysis relies on the functions available in the **R** package archiDART (Delory et al., 2016, 2018). In the first pipeline, traits computed at the root system level (e.g., total root system length, number of roots per branching order) are compared between all simulated and reference root systems. This comparison takes place in three steps: (1) identifying the key morphological, architectural, and topological (Fitter indices, Fitter, 1987; Fitter and Stickland, 1991) traits explaining differences between simulated and reference root systems using multivariate data analysis techniques (e.g., discriminant analysis and principal component analysis), (2) looking at the point in time, beyond the time period for which there are measurements, when simulated and reference root systems start to diverge/converge with regard to the key root system traits identified in the previous step and how large these differences are, and (3) assess the degree of dissimilarity between simulated and reference root systems using dissimilarity metrics based on the raw data (Janssen and Heuberger, 1995).

In the second pipeline, dissimilarities in architecture between reference and simulated root systems are compared using persistent homology. Persistent homology is a topological framework that has proven to be a very powerful tool for capturing variations in plant morphology at different spatial scales (Li et al., 2017, 2018). The main output of a persistent homology analysis is a persistence barcode recording the appearance and disappearance of each root branch when a distance function traverses the branching structure (see Figure 1 in Delory et al., 2018). The degree of similarity between different root system topologies can be assessed by computing a pairwise distance matrix to compare persistence barcodes. In addition, Delory et al. (2018) showed that both trait-based and persistent homology approaches nicely complement each other and allow root researchers to more accurately describe differences in root system architecture (Delory et al., 2018). In our data analysis pipeline, a persistent homology analysis comprises the following steps: (1) computing a persistence barcode for each simulated and reference root system using a geodesic distance function, (2) computing dissimilarities between persistence barcodes using a bottleneck distance, (3) visualize dissimilarities between root systems using multidimensional scaling, and (4) test specific hypotheses using permutational multivariate analysis of variance (PERMANOVA) (Anderson, 2001).

##### 2.2.1.2. M1.2 Long model simulations

In this benchmark, modelers use the same input parameter set as in M1.1, but simulate root system growth and development for a longer time period (60 days). The aim of this second benchmarking exercise is to assess if the different models diverge (or converge) if simulations are run for a longer time period and extrapolate beyond the provided data set (Figure 2B). This is of great importance, as parameterization of RSA models is often based on relatively young plants, whereas knowledge of RSA of older root systems is scarce. Therefore, for this M1.2 scenario, experimental data are not used as the basis of comparison anymore. It has to be noted that these two benchmark problems focus on root architecture dynamics modeling only, thus effect of soil properties on root growth is not explicitly modeled.

*2.2.1.2.1. Required output*. The following results are to be uploaded via pull requests to this path on the github repository: M1 Root architecture development/M1.2 RSA simulation/M1.2 Numerical results.

1. A text file including the model input parameters used for the specific simulator.
2. Simulation output from running the root architecture model using this parameter set in RSML format. Due to the stochastic nature of root architecture models, 100 realizations of each model setup are requested. The file format should be RSML and the file name should be of the form “modelname_replicate,” e.g., “CRootBox_1.rsml.”

*2.2.1.2.2. Analysis pipeline for M1.2*. For the second benchmark (M1.2), three data analysis pipelines are used to compare simulation outputs given by different root architecture models. For this benchmark, the reference experimental data cannot be used as a reference as data of 60 days old plants is not available. The first two data analysis pipelines for M1.2 are very similar to the ones described earlier for the M1.1 benchmark. First, model outputs are compared using morphological, architectural, and topological traits computed at the root system level. Second, differences in root system morphology are analyzed using persistent homology. In addition to these two analysis pipelines, we included a third one to analyse differences in vertical root distribution between root systems simulated with different root architecture models. To do so, we use the modeling approach described in Oram et al. (2018). Briefly, relative cumulative root length density [Y(d)] is computed using Equation (1).

(1) $$\begin{array}{l} Y\left(d\right)=\overset{i=d}{\underset{i=0}{\sum }}RLD\left(i\right)/\overset{\infty }{\underset{i=0}{\sum }}RLD\left(i\right) \end{array}$$

Equation (2) is fitted to the computed Y(d) using a non-linear least square means fitting procedure. The fitting constant β is used to compare modeled rooting depth, with high β corresponding to deep rooting.

(2) $$\begin{array}{l} Y\left(d\right)=1-\beta^{d}, \end{array}$$

#### 2.2.2. Module 2: Water Flow in Soil Only

In this module, we describe benchmark problems that only relate to water flow in soil. Water flow in soil is most commonly described by the Richards equation in three dimensions:

(3) $$\frac{\partial \theta }{\partial t}=\nabla \cdot (K(\theta )(\nabla \psi_{s}+e_{3})),$$

where θ is the volumetric soil water content (cm^3^cm^−3^), *K* is the hydraulic conductivity (cm day^−1^), ψ_*s*_ is the soil water pressure head (cm), and **e_3_** = (0, 0, 1) is the standard unit vector.

The relationship between soil water pressure head and water content is generally described by the water retention curve. In the following we will use the van Genuchten equation (Van Genuchten, 1980) to describe this curve specifying the soil moisture characteristic of specific soils. All participating simulators will solve the exact same equation (i.e., Equation 3), with the same initial and boundary conditions. Therefore, differences between the outputs of different simulators are numerical solution-level differences, i.e., due to numerical scheme and implementation. Different numerical solutions of the Richards equation have been analyzed before, and for some settings analytic solutions exist. We will use the benchmarks presented by Vanderborght et al. (2005) to benchmark the part of the participating functional structural root architecture models where water movement in soil is described. The analytical solutions provided in that paper are related to vertical changes in the soil profile only. As most functional-structural root architecture models have a 3D soil module, they will prescribe no-flux boundary conditions at the sides of a domain with 25 cm length and width for the numerical implementation of those problems.

In the following we will describe the benchmarks for water movement in soil. Table 3 gives an overview of the soil hydraulic properties that will be used throughout all the benchmarks involving water flow in soil.

**Table 3:** Soil hydraulic taken from Vanderborght et al. (2005).

**Soil type**	**θ_*res*_**	**θ_*sat*_**	**α**	**n**	**K_***s***_**	**λ**
	**(–)**	**(–)**	**(cm^**−1**^)**	**(–)**	**(cm d^**−1**^)**	**(–)**
Sand	0.045	0.43	0.15	3.0	1000	0.5
Loam	0.08	0.43	0.04	1.6	50	0.5
Clay	0.1	0.40	0.01	1.1	10	0.5

*θ_res_ is the residual water content, θ_sat_ is the saturated water content, α and n are the van Genuchten parameters, K_sat_ is the saturated soil hydraulic conductivity and λ is the van Genuchten-Mualem parameter*.

##### 2.2.2.1. M2.1: Infiltration

This benchmark scenario is taken from Vanderborght et al. (2005). All parameters, initial and boundary conditions are given in Table 2 and are described below. For each of the soil types, sand, loam and clay, we consider the rate of infiltration into a soil with an initial homogeneous soil water pressure head of ψ_*s*_ = −400 cm. All profiles are 200 cm deep, at the top boundary we prescribe a constant influx of 100 cm d^−1^ as long as the soil is still unsaturated, and a Dirichlet boundary condition of ψ_*s*_ = 0 cm as soon as the soil is fully saturated. Note that the prescribed infiltration value is high, such that in most scenarios, the boundary condition will switch to Dirichlet very soon. At the bottom boundary, we prescribe free drainage. Since this problem only produces gradients in the vertical direction, we compare numerical model results with the 1D analytical solution described in Vanderborght et al. (2005).

**Figure 3:**
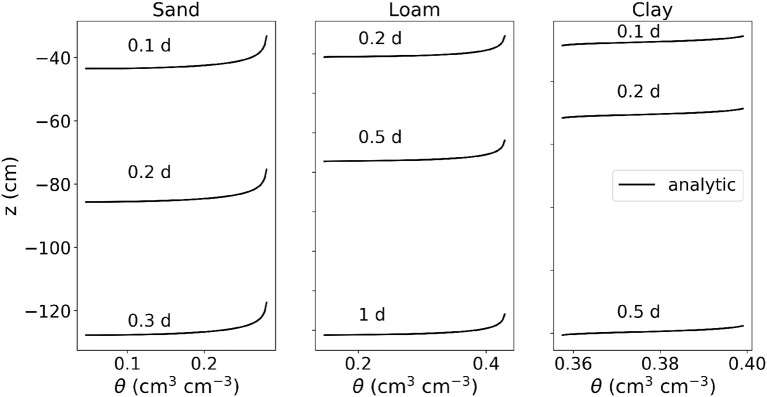
Results of M2.1: Infiltration into three initially dry soils: sand, loam, and clay.

*2.2.2.1.1. Reference solution*. The analytical solution is given by the traveling wave equation

(4) $$\begin{array}{l} \Delta \eta \left(\theta \right)=\eta \left(\theta \right)-\eta \left(\theta_{a}\right)=\left(\theta_{sur}-\theta_{i}\right)\\ \begin{array}{c} \;\int_{\theta }^{\theta_{a}}\frac{D_{w}\left(\theta \right)d\theta }{\left[K\left(\theta_{sur}\right)-K\left(\theta_{i}\right)\right]\left(\theta -\theta_{i}\right)-\left[K\left(\theta \right)-K\left(\theta_{i}\right)\right]\left(\theta_{sur}-\theta_{i}\right)}, \end{array} \end{array}$$

where *D*_*w*_ is the water diffusivity (defined as $D_{w}=K\left(\theta \right)\frac{\partial \psi_{s}}{\partial \theta }$), θ_*sur*_ is the water content at the soil surface, θ_*i*_ is the initial water content, θ_*a*_ is a reference water content (taken to be θ_*a*_ = (θ_*sur*_ + θ_*i*_)/2), $\eta =|z|-\frac{\left[K\left(\theta_{sur}\right)-K\left(\theta_{i}\right)\right]t}{\theta_{sur}-\theta_{i}}$ and Δη(θ) is the distance of the front to the position of the reference water content. The implementation of this analytical solution, implemented in the Jupyter Notebook M2.1 Benchmark problem.ipynb, reproduces Figures 4a–c from Vanderborght et al. (2005), where the water content is plotted after 0.1, 0.2, and 0.3 days for the sand scenario; 0.2, 0.5, and 1 days for the loam scenario; and 0.1, 0.2, and 0.5 days for the clay scenario (see Figure 3).

*2.2.2.1.2. Required output*. The following simulation results of participating models are to be uploaded via pull requests to this path on the github repository: M2 Water flow in soil/M2.1 Infiltration/M2.1 Numerical results.

1. A text file consisting of nine pairs of rows containing comma separated depth values (cm) in the first, and water content (cm^3^cm^−3^) in the second row of the pair. The first three pairs represent the three time points for the sand scenario, the second three pairs represent the three time points of the loam scenario, and the last three pairs represent the three time points for the clay scenario. The file name should be of the form “simulatorname.txt,” e.g., “DuMux.txt.”

Note that we do not prescribe spatial or temporal resolution of the outputs, as that may depend on the individual numerical schemes.

##### 2.2.2.2. M2.2: Evaporation

This benchmark reproduces Figure 5 of Vanderborght et al. (2005). We consider four scenarios (sand, loam 1, loam 2, clay) in which we are interested in the actual evaporation over time from an initially moist soil (ψ_*i*_ = −40 cm for the sand scenario and ψ_*i*_ = −200 cm for all other scenarios). The domain is 100 cm deep with a width and length of 10 cm. At the top boundary, we prescribe a constant efflux of *J*_*s,pot*_ = 0.1 cm d^−1^ for the sand and loam 1 scenario, and 0.3 cm/day for the loam 2 and clay scenarios, at the bottom we prescribe zero-flux. When the soil reaches a critical soil water pressure head of −10,000 cm at the surface, we switch to a Dirichlet boundary condition with ψ_*s*_ = −10,000 cm.

**Figure 4:**
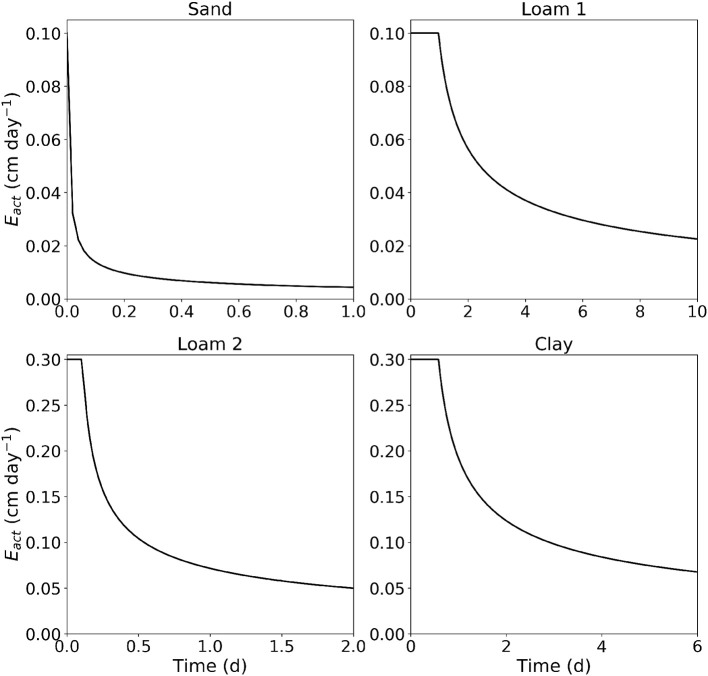
Results of M2.2: Rate of evaporation with respect to time from sand with *J*_*s,pot*_ = 0 1 cm/d, loam with *J*_*s,pot*_ = 0 1 cm/d, loam with *J*_*s,pot*_ = 0 3 cm/d, and clay with *J*_*s,pot*_ = 0 3 cm/d.

*2.2.2.2.1. Reference solution*. The analytical solution to this problem is given by

(5) $$J_{s}(z=0,t)=\{\begin{array}{l} J_{s,pot}\text{ for }t<t_{pot}\\ \frac{S_{w}(\theta_{sur},\theta_{i})}{2\sqrt{t^{\prime }+t-t_{pot}}}\text{ for }t\geq t_{pot} \end{array}$$

where $t^{\prime }=\frac{S_{w}^{2}\left(\theta_{sur},\theta_{i}\right)}{4J_{wpot}^{2}}$, $t_{pot}=\frac{S_{w}^{2}\left(\theta_{sur},\theta_{i}\right)}{2J_{wpot}^{2}}$, $S_{w}\left(\theta_{i},\theta_{sur}\right)=\left(\theta_{i}-\theta_{sur}\right)\sqrt{\frac{4}{\mu }\overset{1}{\underset{0}{\int }}D_{w}\left(\Theta \right)d\Theta }$, $\Theta =|\frac{\theta -\theta_{sur}}{\theta_{i}-\theta_{sur}}|$, $\mu =\frac{3\beta \left(1+{\left\{1-\frac{14}{9}\left[1-\frac{\alpha }{{\left(1-\beta \right)}^{2}}\right]\right\}}^{0.5}\right)}{2\left(1-\beta \right)\left[\frac{\alpha }{{\left(1-\beta \right)}^{2}}-1\right]}$, $\alpha =\frac{\int_{0}^{1}{\left(1-\beta \Theta \right)}^{2}D_{w}\left(\Theta \right)d\Theta }{\int_{0}^{1}D_{w}\left(\Theta \right)d\Theta }$, and $\beta ={\left[\frac{\int_{0}^{1}\Theta D_{w}\left(\Theta \right)d\Theta }{\int_{0}^{1}D_{w}\left(\Theta \right)d\Theta }\right]}^{2}$. Figure 4 shows the rate of evaporation over time for the four scenarios soil, loam 1, loam 2, clay.

*2.2.2.2.2. Required output*. The following simulation results of participating models are to be uploaded via pull requests to this path on the github repository: M2 Water flow in soil/M2.2 Evaporation/M2.2 Numerical results.

1. A text file consisting of two rows containing comma separated depth values (cm) in the first, and root pressure head (cm) in the second for each scenario [i.e., 4 (scenarios) × 2 (rows) = 8 rows]. The file name should be of the form “simulatorname.txt,” e.g., “DuMux.txt.”

Note that we do not prescribe spatial or temporal resolution of the outputs, as that may depend on the individual numerical schemes. It is the responsibility of each participant, to upload the best possible solution.

#### 2.2.3. Module 3: Water Flow in Roots

In this benchmark, we consider water flow in xylem with constant and homogeneous soil water pressure head. This problem is well-described, e.g., in Doussan et al. (1998) and Roose and Fowler (2004). Its analytical solution for a single root was already derived by Landsberg and Fowkes (1978). In Appendix A, we present a derivation that is equivalent to the solution of Landsberg and Fowkes (1978) but uses exponential instead of hyperbolic functions. Briefly, conservation of mass in a branched root network with both axial and radial water flow, neglecting plant water storage and osmotic potential, yields Equation (6),

(6) $$\begin{array}{l} 2r_{root}\pi k_{r}\left(\psi_{s}-\psi_{x}\right)=-k_{x}\frac{\partial^{2}\psi_{x}}{\partial \zeta^{2}}, \end{array}$$

where *r*_*root*_ is the root radius (cm), *k*_*r*_ is the radial conductivity (d^−1^), ψ_*s*_ is the soil water pressure head of the surrounding soil (cm), ψ_*x*_ is the root water pressure head inside the xylem (cm), *k*_*x*_ is the axial conductance (cm^3^ d^−1^), and ζ is the axial coordinate (cm).

##### 2.2.3.1. M3.1: A single root in static soil with constant root hydraulic properties

In this benchmark problem, we assume a vertical single straight root segment surrounded by a soil with a constant and uniform soil water pressure head (i.e., the soil is not in hydrostatic equilibrium). We prescribe the root water pressure head at the root collar as ψ_*x*_|_collar_ = ψ_0_, and no axial flow at the root tips.

**Figure 5:**
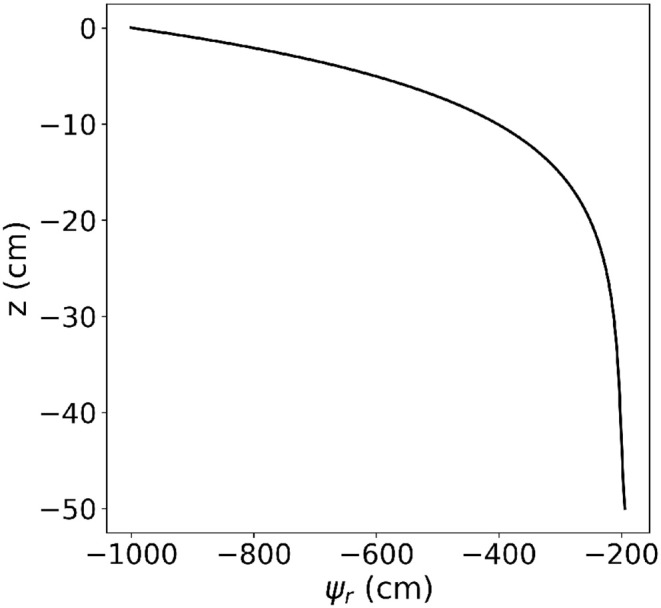
Results of M3.1: Root water pressure head distribution within a single vertical root.

*2.2.3.1.1. Reference solution*. For constant *k*_*r*_ and *k*_*x*_ we can solve Equation (6) yielding

(7) $$\begin{array}{l} \psi_{x}\left(\zeta \right)=\psi_{s}+d_{1}e^{\sqrt{c}\zeta }+d_{2}e^{-\sqrt{c}\zeta }, \end{array}$$

with *c* = 2*r*_*root*_π*k*_*r*_/*k*_*x*_. The integration constants *d*_1_ and *d*_2_ for above boundary conditions are given by

(8) $$\begin{array}{l} d_{1}=d^{-1}\left(e^{-\sqrt{c}l_{seg}}\left(\psi_{0}-\psi_{s}\right)+1\right) \end{array}$$

(9) $$\begin{array}{l} d_{2}=-d^{-1}\left(e^{\sqrt{c}l_{seg}}\left(\psi_{0}-\psi_{s}\right)+1\right), \end{array}$$

where *l*_*seg*_ is the segment length, and *d* is the determinant of above matrix

(10) $$\begin{array}{l} d=e^{-\sqrt{c}l_{seg}}-e^{\sqrt{c}l_{seg}}, \end{array}$$

see Appendix A. Figure 5 shows the analytical solution to this benchmark using the parameters given in Table 4.

**Table 4:** Parameters of scenario M3.1.

l	50	Length of a single straight root (cm)
r_*root*_	0.02	Radius (cm)
k_*z*_	4.32 × 10^−2^	Axial conductivity (cm^3^ d^−1^)
k_*r*_	1.73 × 10^−4^	Radial conductivity (d^−1^)
ψ_*s*_	−200	Static soil water pressure head (cm)
ψ_0_	−1,000	Dirichlet boundary conditions at the root collar (cm)

*2.2.3.1.2. Required output*. The following simulation results of participating models are to be uploaded via pull requests to this path on the github repository: M3 Water flow in roots/M3.1 Single root/M31 Numerical results/.

1. A text file consisting of two rows containing comma separated depth values (cm) in the first, and root pressure head (cm) in the second. The file name should be of the form “simulatorname.txt,” e.g., “DuMux.txt.”

Note that we do not prescribe spatial resolution of the outputs, as that may depend on the individual numerical schemes.

##### 2.2.3.2. Benchmark M3.2: A small root system in a static soil

**Figure 6:**
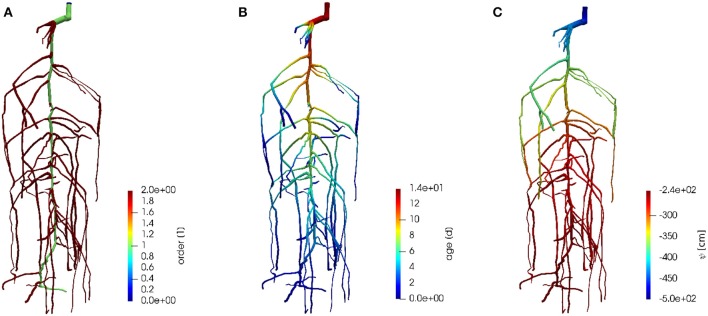
Visualization of the root system of M3.2 with colors denoting **(A)** root order, **(B)** root segment age, **(C)** root water pressure head.

In the following benchmark, we extend benchmark M3.1 from a single root to a root system. We consider water flow inside a small static root system of a lupine plant which was grown for 14 days in a soil-filled column of 20 cm depth and 7 cm diameter. The root system was imaged by MRI at Forschungszentrum Jülich; the segmented root structure is provided in RSML, DGF (Dune grid format) (Bastian et al., 2008) and RSWMS (Javaux et al., 2008) formats in the folder M3 Water flow in roots/M3.2 Root system/root_grid on the github repository. It is visualized in Figures 6A,B with colors denoting root order and root segment age.

*2.2.3.2.1. Reference solution*. The reference solution for this problem is given by the hybrid analytical-numerical solution of water flow in the root hydraulic architecture proposed by Meunier et al. (2017). The advantage of this solution is that it is independent of the spatial resolution of the root system (i.e., root segment length).

**Figure 7:**
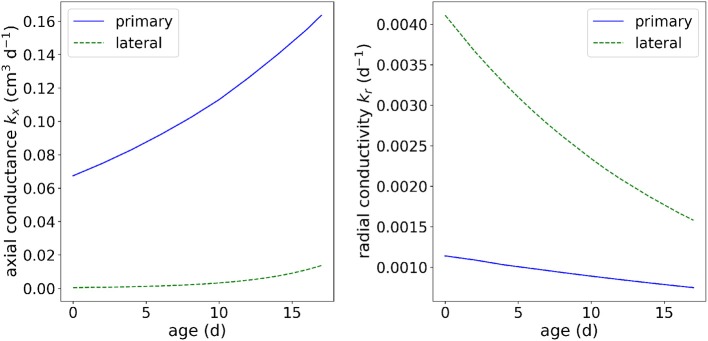
Root hydraulic properties dependency on root type and root segment age.

We consider two scenarios. The first one uses the same constant root hydraulic properties as given in Table 4, i.e., considering the same root hydraulic properties for each root segment. In the second scenario, we consider age-dependent root hydraulic properties for tap root and laterals of lupine as obtained by Zarebanadkouki et al. (2016, exponential function scenario) and converting distance from root tip to root age by assuming a root growth rate of 1 cm d^−1^. This parameterization takes into account that roots get a higher axial conductivity and lower radial conductivity as they are becoming older (see Figure 7, a table with the actual values is provided on the github repository, in: M3 Water flow in roots/M3.2 Root system/M3.2 Benchmark problem.ipynb.

**Figure 8:**
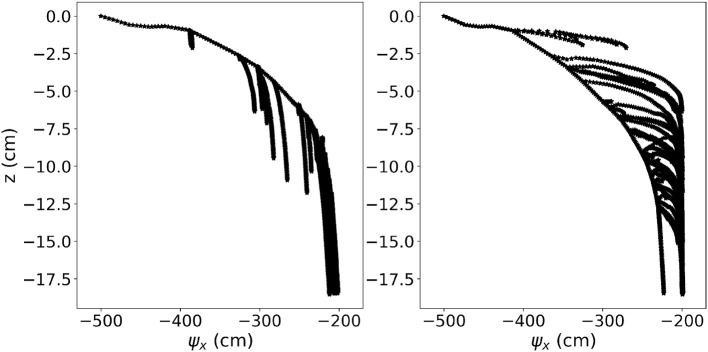
Results of M3.2. **(Left)** Xylem pressure in each root segment of a root system with constant hydraulic properties. **(Right)** Xylem pressure in each root segment of a root system with age-dependent hydraulic properties.

A sample 3-D visualization of the model output is shown in Figure 6C for the constant root hydraulic properties scenario. Figure 8 shows the effect of constant and age-dependent root hydraulic properties under otherwise same (soil and boundary) conditions.

*2.2.3.2.2. Required output*. The following simulation results of participating models are to be uploaded via pull requests to this path on the github repository: M3 Water flow in roots/M3.2 Root system/M32a Numerical results and M3 Water flow in roots/M3.2 Root system/M32b Numerical results for the constant and age-dependent root hydraulic properties cases.

1. A text file consisting of two rows containing comma separated depth values (cm) in the first, and root pressure head (cm) in the second. The file name should be of the form “simulatorname.txt,” e.g., “DuMux.txt.”

Note that we do not prescribe spatial resolution of the outputs, as that may depend on the individual numerical schemes.

#### 2.2.4. Coupled Benchmark Scenarios C1: Root Water Uptake by a Static Root System

The way of coupling can easily introduce differences in simulated results because of numerical errors (especially when there is two way coupling) or because different assumption are made when implementing the coupling. No analytical solutions exists for the coupled problems presented here, but the coupling (C) benchmarks are intended to quantify differences between model outputs of coupled models. We may see differences observed in the non-coupled benchmarks to be amplified, or to be irrelevant for the coupled problem.

##### 2.2.4.1. C1.1: Water uptake by a single root

This benchmark follows the paper of Schröder et al. (2008). Here we aim to see to what extent the different participating models can reproduce the hydraulic conductivity drop near the root surface under different soil conditions and transpiration demands. Thus, it requires the participating line-source based models to strongly increase the spatial resolution of the 3D soil domain. From this benchmark, we will learn, whether the spatial resolution required to reproduce radial soil water pressure head gradients would be in a feasible order of magnitude for larger soil-root systems or not. If not, there are approaches to estimate soil water pressure head drop at the root-soil interface from bulk soil values as e.g., in Schröder et al. (2009), Beudez et al. (2013), and Mai et al. (2019), see also benchmark C1.2.

##### 2.2.4.2. Reference solution

The analytical solution is based on the analytical solutions of the 1D radially symmetric problem of water uptake by a single root, in which root water uptake is described as a boundary condition at the root-soil interface. We consider here two water uptake regimes, a non-stressed condition with maximum root uptake (*q*_*root*_), and a stressed condition with a limiting plant root water potential constraining uptake. Based on the steady-rate assumption and using the matric flux potential $\Phi \left(h_{c}\right)=\int_{-\infty }^{h_{c}}K\left(h\right)dh$ that linearizes the Richards equation, the radial soil water pressure head profiles for non-stressed and stressed conditions (stress conditions are given when the soil water pressure head at the root surface reaches −15,000 cm) are given by

(11) $$\begin{array}{l} \Phi_{nostress}\left(r\right)=\Phi_{r_{out}}+\left(q_{root}r_{root}-q_{out}r_{out}\right)\\ \;[\frac{r^{2}/r_{root}^{2}}{2\left(1-\rho^{2}\right)}+\frac{\rho^{2}}{1-\rho^{2}}\left(\text{ln}\frac{r_{out}}{r}-\frac{1}{2}\right)]\\ \begin{array}{c} \;+\;q_{out}r_{out}\text{ln}\frac{r}{r_{out}} \end{array} \end{array}$$

and

(12) $$\begin{array}{l} \Phi_{stress}\left(r\right)=\left(\Phi_{r_{out}}-\Phi_{r_{root}}+q_{out}r_{out}\text{ln}\frac{1}{\rho }\right)\\ \begin{array}{c} \;\frac{r^{2}/r_{root}^{2}-1+2\rho^{2}\text{ln}r_{root}/r}{\rho^{2}-1+2\rho^{2}ln1/\rho }+q_{out}r_{out}\text{ln}\frac{r}{r_{root}}+\Phi_{root}, \end{array} \end{array}$$

where $\rho =\frac{r_{out}}{r_{root}}$.

Given the soil water pressure head at the outer boundary, the solution computes the soil water pressure head profile toward the root. Due to the steady-rate assumption, the problem has become a stationary boundary value problem. However, under non-stressed conditions, we can calculate the time that corresponds to a given radial soil water pressure head profile by dividing the volume of water removed from the soil domain by the known water flow rate. The water remaining in a 1 cm long hollow cylinder around the root is given by

(13) $$\begin{array}{l} V=\int_{0}^{2\pi }\int_{r_{root}}^{r_{out}}r\theta drd\phi =2\pi \int_{r_{root}}^{r_{out}}r\theta dr, \end{array}$$

θ being the water content. The initially available water volume in the soil domain is given by

(14) $$\begin{array}{l} V_{i}=\left(r_{out}^{2}-r_{root}^{2}\right)\pi \theta_{i}. \end{array}$$

Thus, until the onset of stress, the corresponding time at which a given radial profile is reached is given by

(15) $$\begin{array}{l} t=\frac{\left(V_{i}-V\right)}{2r_{root}\pi q_{root}}. \end{array}$$

**Figure 9:**
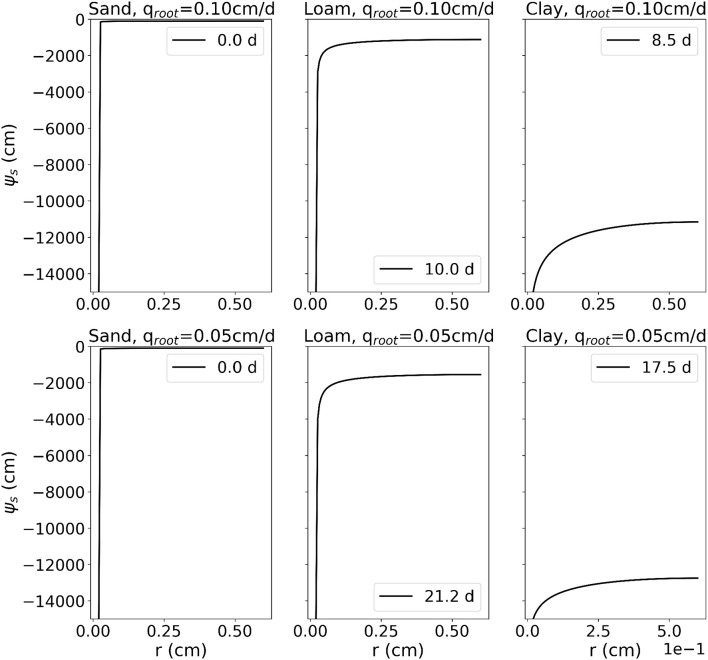
Results of C1.1: Soil water pressure head gradients around a single, transpiring, root at the onset of stress and the time of its occurrence.

For the three soils sand, loam, and clay (Table 3), we compute the analytical solution for two different values of *q*_*root*_ (*q*_*root*_ = 0.1 cm/d and *q*_*root*_ = 0.05 cm/d, alternatively), and with the following parameters: *r*_*root*_ = 0 02 cm, *r*_*out*_ = 1 cm, ψ_*s,lim*_ = −15,000 cm, *q*_*out*_ = 0.0 cm/d, for different soil water pressure heads at the outer end of the cylinder. Figure 9 shows the soil water pressure head gradients at the onset of stress (i.e., when the soil water pressure head at the root surface reached −15,000 cm) and the time of its occurrence. The value of the initial soil water pressure head is taken to be ψ_*s,i*_ = −100 cm. This analytical solution is for radial water flow in soil toward the root only, i.e., not considering gravity or water flow inside the roots. Ideally, in their numerical implementation of this benchmark, the different participating models will turn off gravity effects. The soil domain for this numerical implementation has a size of *l* × *w* × *d* = 1 × 1 × 1 cm. The horizontal spatial resolution is high enough such that hydraulic conductivity drop near root surface can be resolved. The axial and radial conductances are high, such that the pressure inside the root is everywhere the same and the uptake flux is uniform.

*2.2.4.2.1. Required output*. The following simulation results of participating models are to be uploaded via pull requests to this path on the github repository: M3 Water flow in roots/M3.2 Root system/M32a Numerical results and M3 Water flow in roots/M3.2 Root system/M32b Numerical results for the constant and age-dependent root hydraulic properties cases.

1. A text file consisting of two rows containing comma separated radial distances from the root surface (cm) in the first, and soil pressure head (cm) in the second for each soil and transpiration rate scenario [i.e., 3 (soils) × 2 (transpiration rates) × 2 = 12 rows]. The file name should be of the form “simulatorname.txt,” e.g., “DuMux.txt.”

Note that we do not prescribe spatial or temporal resolution of the outputs, as that may depend on the individual numerical schemes.

#### 2.2.5. C1.2: Water Uptake by a Root System From Drying Soil

This benchmark scenario considers water uptake by a static 8-days-old lupine root system given in the public data set (Koch, 2019) as RSML or DGF. The root is the same as the one in benchmark M3.2, only younger, in order to reduce the computational cost for the reference scenario. The root system has been segmented from MRI measurements. The lupine is embedded in a soil box of *l* × *w* × *d* = 8 × 8 × 15 cm filled with loam (soil hydraulic properties given in Table 3). The benchmark is to evaluate the accuracy of root water uptake models under conditions of drying soil. To this end, the soil has an initial water content of θ_top_ = 0.129, corresponding to a pressure head ψ_*s*, top_ = −659.8 cm at the soil surface (*z* = 0). The pressure head in the rest of the domain initially follows a hydrostatic distribution

(16) $$\begin{array}{l} \psi_{s,\text{i}}=\psi_{s,\text{top}}-z, \end{array}$$

where *z* (in cm) denotes the vertical position (upward-pointing axis, zero at soil surface). At all soil boundaries, as well as at the root tips, no-flux boundaries are prescribed. A potential transpiration rate is given as the sinusoidal diurnal function

(17) $$\begin{array}{l} Q_{\text{pot}}\left(t\right)=\bar{Q}\left[1+\text{sin}\left(2\pi t-\frac{\pi }{2}\right)\right], \end{array}$$

where the mean transpiration rate is $\bar{Q}=$ 6.4^3^ cm d^−1^, the time *t* is given in days, and *Q*_pot_(*t* = 0) = 0, that is, the simulation starts at night. The potential transpiration rate *Q*_pot_, Equation (17), is enforced at the root collar (Neumann boundary condition) as long as the root water pressure head at the root collar is above ψ_*x*,crit_ = −15, 290 cm (corresponding to −1.5 MPa). If this critical root water pressure head at the root collar is reached, the boundary condition is switched to a Dirichlet type boundary condition, enforcing a constant pressure head ψ_*x*,crit_ = −15,290 cm at the root collar. This informal description is intentional, as the actual implementation of such a boundary condition may vary from simulator to simulator. We consider two scenarios. In scenario C1.2a the root hydraulic properties are constant. The tap root and lateral root conductivities are $k_{x}=4.32\times 10^{-23}$ cm d^−1^ and $k_{r}=1.73\times 10^{-4}$ d^−1^ (Table 4). For scenario C1.2b the root hydraulic properties depend on the root type and root age and are shown in Figure 7.

Given the soil domain Ω and the network of root center-lines Λ, we solve the following coupled system of equations

(18) $$\begin{array}{l} \frac{\partial \theta }{\partial t}-\nabla \cdot \left(K\left(\theta \right)\left(\nabla \psi_{s}+e_{3}\right)\right)=q\left(\psi_{x},\psi_{s}\right)\;\text{in }\Omega , \end{array}$$

(19) $$\begin{array}{l} -\frac{\partial }{\partial \zeta }\left(k_{x}\frac{\partial \psi_{x}}{\partial \zeta }+\frac{\partial z}{\partial \zeta }\right)=\hat{q}\left(\psi_{x},\psi_{s}\right)\;\text{on }\Lambda , \end{array}$$

subject to the boundary conditions specified above, where ζ is a scalar parameterization (local axial coordinate) of the root segments. The specific radial flux $\hat{q}$ in units (cm2 d-1) is given by the difference in the average soil water pressure head on the root surface and in the xylem multiplied by the root radial conductivity. The formulation of *q* in Equation (18) may be different between different participating models. A discussion on singularity issues when evaluating the soil water content at the root center line can be found in Koch et al. (2018b). In many cases, the soil discretization is much larger than the root diameter, and thus the drop in hydraulic conductivity near the root surface in dry soils may not be sufficiently resolved in the soil domain. Different approaches for the determination of the sink term for root water uptake are likely to differ most in dry soil. The reference solution to this benchmark is designed to evaluate possible differences between the models in that regard.

#### 2.2.6. Reference solution

As no analytical solutions exist for this problem of coupled water flow in the soil-root system, we designed a reference solution with a numerical model that explicitly considers the physical presence of roots in the soil domain, i.e., the soil mesh is highly refined around all roots and water uptake is modeled via boundary conditions at all the root surfaces. Thus, this reference solution does not make any assumptions that are inherent in the definition of the sink terms for root water uptake in the line source-based models. An explicit 3D soil grid is also used in Daly et al. (2018). However here, the soil is additionally coupled to the xylem flow in the root. The root is still modeled as a network of one-dimensional segments (center-line representation). Each segment has a specific radius as specified in the RSML grid file to this benchmark. A three-dimensional representation of the root system is implicitly given by the union of all spheres along the root center-lines. Using this implicit representation a soil grid excluding the root system was generated using the C++ geometry library CGAL (The CGAL Project, 2019). In order to reduce the number of vertices in the mesh, the mesh is locally refined around the root-soil interface. The resulting mesh is available in the Gmsh format (Geuzaine and Remacle, 2009) in the data set. For the evaluation of the radial flux, which is a coupling condition on the soil faces σ representing the root-soil interface, we integrate over each face

(20) $$\begin{array}{l} F_{r}=\int_{\sigma }r_{\text{root}}k_{r}\left(\psi_{s}-\psi_{x}\right)\text{d}A. \end{array}$$

While the soil water pressure head is defined on the face, the corresponding root xylem water pressure head has to be found by a mapping. To this end the integration point is first mapped onto the root surface using its implicit representation. Then the point is mapped onto the corresponding root center-line (a line segment) by finding the closest point on the line segment. There, ψ_*x*_ is evaluated. The flux is added as a source term in the corresponding segment in the root. The model is implemented in the open-source porous media simulator DuMu^x^ (Flemisch et al., 2011; Koch et al., 2018a; Koch et al., 2019). The coupled system is solved with a fully coupled manner, using Newton's method, and monolithic linear solver (block-preconditioned stabilized bi-conjugate gradient solver) in each Newton iteration. The equations are discretized in time with an implicit Euler method, and in space with a locally mass conservative vertex-centered finite volume method (BOX method Helmig, 1997). The maximum time step size is Δ*t* = 1,200 s. The actual time step size may be sometimes chosen smaller, depending on the convergence speed of the Newton method. Output files are produced in regular intervals every 1,200 s starting with the initial solution. The simulation time is 3 d.

**Figure 10:**
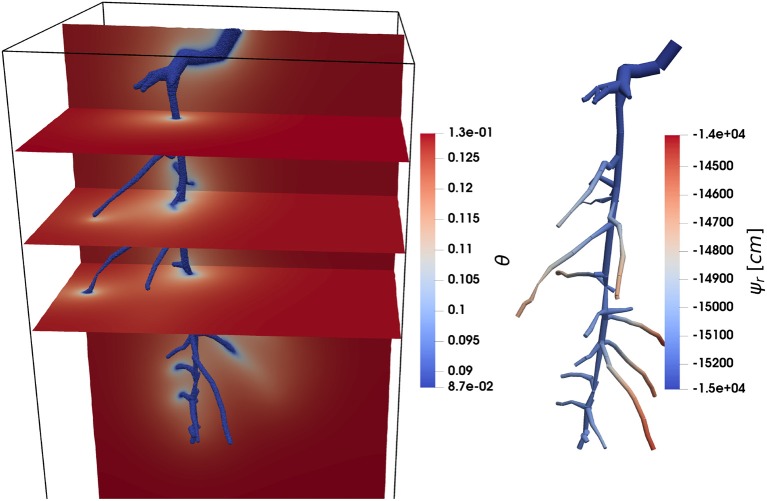
C1.2: Root water uptake by a static root system over time. **(Left)** Visualization of the volumetric soil water content on vertical and horizontal slices through the soil domain and along the root surfaces. **(Right)** Root water pressure head.

**Figure 11:**
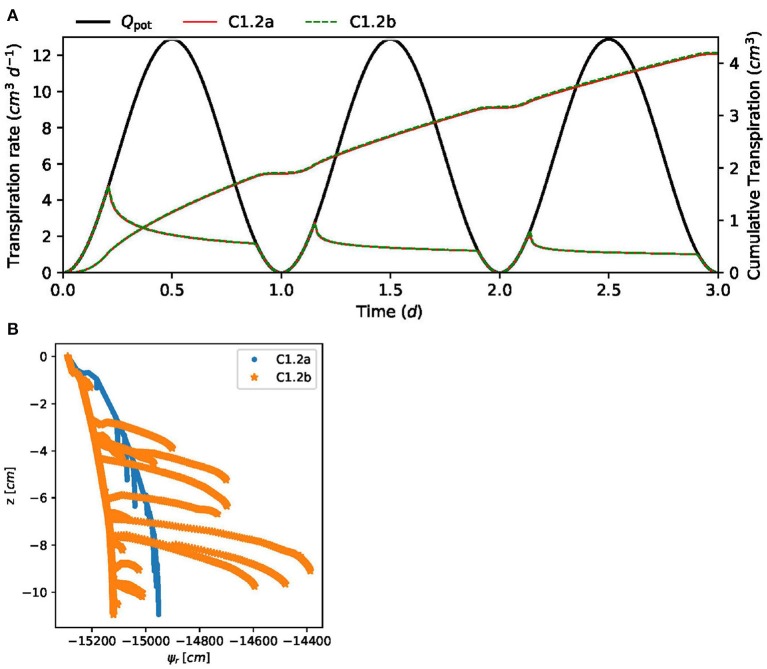
Results of C1.2 for two scenarios, constant and age-dependent root hydraulic properties. **(A)** Actual transpiration of reference solution. **(B)** Root water pressure head distributions inside the root system.

Soil water content and root water pressure head in a three-dimensional plot is shown in Figure 10 for C1.2b. Figure 11A shows the potential and actual transpiration rates for both scenarios, with constant and age-dependent root hydraulic properties. The curves hardly differ since the water pressure head drop is dominated by the low conductivity of the dry soil. In Figure 11B, the differences between scenarios are more clearly visible in terms of the minimal and maximal root water pressure head with respect to time.

##### 2.2.6.1. Required outputs

To compare the results between the participating models, the desired outputs are

- VTK files (3D) of soil water pressure head and water content on the first, second, and third day (*t* = 0.5, 1.5, 2.5 d). For output written every 1,200 s this means the output files with the number 36, 108, and 180
- VTK files (lines in 3D) of root water pressure head in the first, second, and third day (*t* = 0.5, 1.5, 2.5 d)
- CSV file with three data points per time step (each 1,200 s starting with *t* = 0): time and actual transpiration rate
- CSV file with three data point per time step: time and minimum and maximum root water pressure head.

File names of the VTK files should indicate the simulator name, the state variable, the domain, and the output time in days, e.g., “DuMux_soil_theta_1d.vtk.” File names of the CSV files should indicate the simulator name and output time it days, e.g., “Dumux_1.csv.”

### 2.3. Coupled Benchmark Scenarios C2: Root Water Uptake by a Dynamic Root System

In this benchmark, we wish to explore differences caused by the approach of root growth modeling. We assess how the differences in root architecture parameters resulting from M1.2 propagate (or not) in the computation of the root water uptake from soil. In this example, we do not consider the effect of soil properties on root growth, but only the differences that arise from the different root systems according to M1.2.

#### 2.3.1. C2.1: Water Uptake by a Single Root

Before looking at the root system, we look at how the implementation of the growth itself affects computed root water uptake for a single root. This scenario is analogous to C1.1, but with a single root growing at an elongation rate of 2 cm/d from 1 to 10 cm length.

##### 2.3.1.1. Required outputs

The required outputs for model intercomparison are

- VTK files of 3D soil water pressure head and water content in soil at a temporal resolution of 1 day up until 60 days (point data)
- VTK files of xylem water pressure head (point data)
- Text files with two lines: time and corresponding actual transpiration.

#### 2.3.2. C2.2: Water Uptake by a Root System

This scenario is the same as C1.2b, but replacing the static root system with a growing root system. The root growth parameters are for each model the results of M1.2; simulations start from a seed and run until a 60 days old root system. The domain size is 25 × 25 × 100 cm, the potential transpiration *Q*_*pot*_ = 0.5 cm^3^ d^−1^ is scaled proportional to the root volume divided by the maximal root volume at maturity.

##### 2.3.2.1. Required outputs

- VTK files of 3D soil water pressure head and water content in soil at a temporal resolution of 1 day up until 60 days (point data)
- VTK files of xylem water pressure head (point data)
- Text files with two lines: time and corresponding actual transpiration.

File names of the VTK files should indicate the simulator name, the state variable, the domain, and the output time in days, e.g., “DuMux_soil_theta_1d.vtk.” File names of the CSV files should indicate the simulator name and output time it days, e.g., “Dumux_1.csv.”

### 2.4. Automated Comparison Within All Benchmark Problems

Each benchmark folder on the github repository contains a Jupyter Notebook named “Automated comparison.” It provides the analytical solution of the respective benchmark and in addition includes Python code that automatically loads all the outputs of participating models that are provided in the “Numerical results” folder of that benchmark. As soon as new outputs are provided, they are automatically included in the analysis. Currently, different model outputs are already available. We envision more participating models' outputs to be provided in this way. Future analysis will include graphical and quantitative approaches.

## 3. Discussion

The benchmark problems considered here cover the basic processes of root water uptake from soil by 3D root architectures and focus on the coupling of root and soil domains. Root water capacity may be important in the case of trees (Janott et al., 2011) or when plants are under extreme water stress (Fang et al., 2019). Cavitation may induce a reduction of the specific root axial conductance (Sperry et al., 2003; Janott et al., 2011; Ahmad et al., 2018). Soil conditions can strongly affect root development (Schnepf et al., 2018b; de Moraes et al., 2019). At a later stage, these processes may be included in the benchmarking initiative by adding suitable benchmarking problems, e.g., including data from field studies, such as that of (de Moraes et al., 2019).

Root water uptake and evapotranspiration are a major factor in larger scale models, such as crop or land surface models (Kimball et al., 2019). They usually consider only the vertical soil dimension, thus have a 1-dimensional soil module. Thus, the functional-structural root architecture models considered here are not directly applicable. However, several examples have shown how the information of the 3-dimensional root hydraulic architecture can be implicitly considered in those models to compute root water uptake from 1D soils (Janott et al., 2011; Couvreur et al., 2014). In analogy to the electric circuit model, Couvreur et al. (2012) introduced a reduction of the 3-dimensional root hydraulic architecture to modular macroscopic equations and parameters operational for land surface and crop models (Baram et al., 2016; Sulis et al., 2019). Such a multiscale approach offers to connect the dots between models and measurable hydraulic and geometrical properties from the cell to the plant scale (Couvreur et al., 2018; Passot et al., 2018; Meunier et al., 2019) thus integrating essential processes and functional-structural properties for large scale models.

Additional processes, such as root water capacity and cavitation or the reduction of considered soil dimensions are out of the scope of this first initiative. However, we hope that it will function as a seed to initialize additional individual studies that consider those processes. We welcome such contributions in the Research Topic “Benchmarking 3D-Models of Root Growth, Architecture and Functioning” of “Frontiers in Plant Science.”

Numerical results of the different simulators will be compared to reference solutions or data where possible. For the root architecture models, measured root systems are available for comparison. The analysis pipelines for the RSA model outputs are outlined for the M1 module. The results of the different RSA simulators will be analyzed using both univariate and multivariate methods on root system traits as well as persistent homology. The soil and root water flow modules M2 and M3 have analytical solutions against which simulator results are compared. For the coupled problem with static root system, we offer a reference solution based on an explicit 3D simulation in which the root volume in the soil domain is accounted for. Quantitative comparison between different simulator results and reference solutions will rely on both residual-based and association-based goodness of fit measures (Bellocchi et al., 2010). The only benchmark problem without reference solution is the coupled problem with dynamic root architecture development, C2. Thus, for this problem the outcomes of the different simulators will be compared with each other. The aim is to obtain information about how diverse the different simulators are, and to quantify how the differences that arise from the RSA model choices (M1), the numerical implementation of soil and root water flow (M2-3) as well as the domain coupling choices (C1) propagate into the root water uptake computations. Based on these quantitative results, model users will be able to decide which model is suitable for a given application.

## 4. Conclusions

Functional-structural root architecture models have been compared qualitatively (e.g., Dunbabin et al., 2013), but until now no quantitative benchmarking existed. In other communities, benchmarking has been done or is ongoing, e.g., AgMIP (Porter et al., 2014) for crop models, CMIP (Eyring et al., 2016) for climate models, subsurface reactive transport models (Steefel et al., 2015). With this paper, we propose a framework for collaborative benchmarking of functional-structural root architecture models that allows quantitative comparison of the outputs of different simulators with reference solutions and with each other. This framework is presented using Jupyter Notebooks. Behind every “module” benchmark, there is a working code that explains and implements the reference solution or analysis of reference data. For both, “module” and “coupled” benchmarks, Jupyter Notebooks facilitate the automated comparison of simulator simulation outputs that are stored in specified folders of a public github repository. In this way, new numerical simulators that may be developed in the future may still be added to the automated comparison. All the analysis that is done in the Jupyter Notebooks is freely available so that the comparisons and analysis of uploaded model outputs will be transparent and repeatable. Future efforts will aim at extending the benchmarks from water flow in root and soil systems to further processes, such as solute transport, rhizodeposition, etc. We expect that this benchmarking will result in a better understanding of the different models and contribute toward improved models, with which we can simulate various scenarios with greater confidence. It will set standards for future model developments, ensuring that bugs, numerical errors or conceptual misunderstandings do not affect the value of future work. This is a step toward developing those models into the much-needed aid in the design of agricultural management schemes and model-guided crop breeding. These models may also be useful in ecology, e.g., to study species complementarity.

## Data Availability Statement

The datasets generated for this study can be found in the github repository https://github.com/RSA-benchmarks/collaborative-comparison.

## Author Contributions

AS initiated this benchmark initiative. AS, CB, VC, BD, CD, AK, TK, MJ, DL, GL, TM, FM, LP, JP, EP, VS, JV, HV, and MW together designed the benchmark problems. AS, VC, BD, AK, TK, MJ, DL, GL, FM, and JP contributed to the implementation of benchmark problems in Python or R. ML provided the MRI measurements of root architecture. All authors have contributed to the writing of the manuscript.

### Conflict of Interest

The authors declare that the research was conducted in the absence of any commercial or financial relationships that could be construed as a potential conflict of interest. The reviewer DH declared a past co-authorship with one of the authors, BD to the handling Editor.

## A. Appendix

### A.1. Derivation of the Analytical Solution of Water Flow Inside the Root System

The axial water flow rate in the xylem *Q*_*x*_ (cm^3^ day^−1^) is given by

(A1) $$Q_{x}=-k_{x}\left(\frac{\partial \psi_{x}}{\partial \zeta }+v\cdot e_{3}\right),$$

where *k*_*x*_ is the axial conductance (cm^3^ day^−1^), ψ_*x*_ is the pressure inside the xylem (cm), ζ is the local axial coordinate **e_3_** the unit vector in *z*-direction, and *v* the normalized direction of the xylem.

The radial water flow rate is given by

(A2) $$\begin{array}{l} Q_{r}=-2r_{root}\pi l_{seg}k_{r}\left(\psi_{s}-\psi_{x}\right) \end{array}$$

with units (cm^3^ day^−1^), where *r*_*root*_ is the root radius (cm), *l*_*seg*_ is the length of each root segment (cm), *k*_*r*_ is the radial conductivity (day^−1^), and ψ_*s*_ is the soil water pressure head of the surrounding soil (cm). The equation is neglecting osmotic potential and is based on Equation (3.3) of Roose and Fowler (2004). Note that around the root a homogeneous soil water pressure head is assumed, therefore there is actually no hydrostatic equilibrium.

For each segment of length *l*_*seg*_ mass conservation yields

(A3) $$\begin{array}{l} 0=Q_{x}{|}_{l_{seg}}-Q_{x}{|}_{0}+Q_{r} \end{array}$$

(A4) $$\begin{array}{l} -\frac{1}{l_{seg}}Q_{r}=-\frac{1}{l_{seg}}\left(Q_{x}{|}_{0}-Q_{x}{|}_{l_{seg}}\right)\text{ and for }l_{seg}\to 0 \end{array}$$

(A5) $$\begin{array}{l} 2r_{root}\pi k_{r}\left(\psi_{s}-\psi_{x}\right)=-k_{x}\frac{\partial^{2}\psi_{x}}{\partial \zeta^{2}} \end{array}$$

see Equation (3.4) of Roose and Fowler (2004), where *v*_3_ is the *z*-component of the normalized xylem direction (cm).

Integrating this ordinary differential equation leads to an explicit equation for ψ_*x*_(ζ)

(A6) $$\begin{array}{l} \psi_{x}\left(\zeta \right)=\psi_{s}+d_{1}e^{\sqrt{c}\zeta }+d_{2}e^{-\sqrt{c}\zeta }, \end{array}$$

where *c*: = 2*aπk*_*r*_/*k*_*x*_, and *d*_1_, and *d*_2_ are integration constants that are derived from the boundary conditions.

To exemplify, we calculate *d*_1_, and *d*_2_ for a Dirichlet boundary condition at the root collar, and no-flux boundary conditions at the tip. The Dirichlet boundary conditions at the collar of the root system ψ_*x*_|_collar_ = ψ_0_ is inserted into the analytic solution (Equation A6), and yields

(A7) $$\begin{array}{l} \psi_{s}+d_{1}+d_{2}=\psi_{0}. \end{array}$$

The Neumann boundary condition *Q*_*x*_|_*l*_*seg*__ = 0 (Equation A2) leads to

(A8) $$\begin{array}{l} \frac{\partial \psi_{x}}{\partial \zeta }{|}_{l_{seg}}=v_{3}, \end{array}$$

where *l*_*seg*_ is the length of the root segment. Using the derivation of the analytical solution yields

(A9) $$\begin{array}{l} d_{1}\sqrt{c}e^{\sqrt{c}l_{seg}}-d_{2}\sqrt{c}e^{-\sqrt{c}l_{seg}}=v_{3}. \end{array}$$

For a straight downward segment *v*_3_ = −1, Equations (A7) and (A9) can be summarized as

(A10) $$\begin{array}{ll} \left(\begin{array}{cc} 1 & 1\\ \sqrt{c}e^{\sqrt{c}l_{seg}} & -\sqrt{c}e^{-\sqrt{c}l_{seg}} \end{array}\right)\left(\begin{array}{c} d_{1}\\ d_{2} \end{array}\right) & =\left(\begin{array}{c} \psi_{0}-\psi_{s}\\ -1 \end{array}\right) \end{array}$$

Solving this linear equation for *d*_1_ an *d*_2_ yields

(A11) $$\begin{array}{l} d_{1}=d^{-1}\left(e^{-\sqrt{c}l_{seg}}\left(\psi_{0}-\psi_{s}\right)+1\right) \end{array}$$

(A12) $$\begin{array}{l} d_{2}=-d^{-1}\left(e^{\sqrt{c}l_{seg}}\left(\psi_{0}-\psi_{s}\right)+1\right), \end{array}$$

where *d* is the determinant of above matrix

(A13) $$\begin{array}{l} d=e^{-\sqrt{c}l_{seg}}-e^{\sqrt{c}l_{seg}}. \end{array}$$
